# Educational Nursing Intervention in Reducing Hospital Readmission and the Mortality of Patients with Heart Failure: A Systematic Review and Meta-Analysis

**DOI:** 10.3390/jcdd9120420

**Published:** 2022-11-28

**Authors:** Cleidinaldo Ribeiro de Goes Marques, Andreia Freire de Menezes, Yasmim Anayr Costa Ferrari, Alan Santos Oliveira, Arthur César Melo Tavares, André Sales Barreto, Rita de Cássia Almeida Vieira, Cassiane Dezoti da Fonseca, Eduesley Santana-Santos

**Affiliations:** 1 Nursing Departament, Universidade Federal de Sergipe, Aracaju 49060-108, Brazil; 2 Nursing Graduate Program, Universidade Federal de Sergipe, Sao Cristovao 49100-000, Brazil; 3 Environmental and Health, Universidade Tiradentes, Aracaju 49065-510, Brazil; 4 Health’s Science Graduate Program, Universidade Federal de Sergipe, Aracaju 49060-108, Brazil; 5 Education in Health Departament, Universidade Federal de Sergipe, Lagarto 49400-000, Brazil; 6 Medical and Surgical Nursing Departament, Federal University of Sergipe, São Paulo 04021-001, Brazil

**Keywords:** heart failure, nursing, patient education, home visit, telephone contact

## Abstract

(1) Background: Heart failure (HF) represents a public health problem due to its high morbidity and mortality, increased consumption of health resources, prolonged hospitalization, and frequent readmissions. This study was conducted to evaluate the effectiveness of a nursing educational intervention using home visits (HV) combined with telephone contact in reducing hospital readmission and the mortality of patients with HF. (2) Methods: This is systematic review and meta-analysis of randomized controlled trials (RCTs). The databases used were CINAHL, Cochrane, PubMed and SciELO. A gray literature search included Google Scholar, OpenThesis, Clinical trials and reference lists of eligible studies. RCTs of patients diagnosed with HF were included, distributed between the control group (CG) and intervention (IG), in which the IG was submitted to the nursing intervention with HV and telephone contact in association and analyzed the result of readmission and mortality. (3) Results: The search resulted in 2528 articles and, after following steps, 11 remained for final analysis. A total of 1417 patients were analyzed and distributed: 683 in the IG and 734 in the CG. As a primary outcome, the meta-analysis identified a 36% reduction in the risk of readmission [RR 0.64, 95% CI, 0.54–0.75, *p* < 0.01] and a 35% reduction in mortality in the IG [RR 0.65, 95% CI, 0.50–0.85, *p* < 0.01]. Heterogeneity was moderate for readmission and homogeneous for mortality. (4) Conclusions: HV and telephone contact are an effective intervention strategy for nurses’ educational practice.

## 1. Introduction

Heart failure (HF) is a complex clinical syndrome characterized by structural and functional cardiac abnormalities that impairs ventricular function of storing or ejecting blood [1]. The disease affects a significant number of people in the world and represents a public health problem due to its high morbidity and mortality (approximately 17 million deaths), increased consumption of health resources, prolonged hospitalization, and frequent readmissions [2].

Constant hospital readmissions have been associated with decompensation of the disease, which results from exacerbation of the signs and symptoms triggered by cardiovascular or non-cardiovascular factors [3]. Therefore, HF management programs constitute a patient care approach to improve adherence to the proposed therapeutic regimen [4].

Outpatient care provided by nurses to patients with HF has been the focus of studies, showing a reduction in hospital readmissions, whether it is performed on a technical (performing health procedures), educational (guidance on self-care) or managerial (referral to complementary health services) [5]. A multidisciplinary team is highly recommended for long-term follow-up of patients with HF, but each country will provide assistance according to its local health system. However, if multidisciplinary monitoring is not possible, the participation of nurses is indicated [6].

Supportive care recommendations of previous studies suggest the use of home visits (HV) between 7–14 days after hospital discharge, with assessment performed by health professionals [2,7]. This educational strategy was tested and has a proven impact in reducing morbidity and mortality [8]. However, some situations may limit the use of HV, such as territorial extension, ability to access the location of the patient, and reduced financial resources [1]. In parallel, telephone monitoring is used as an adjuvant method of follow-up, and data from Azeka and collaborators indicate the efficiency of telephone follow-up when recording data of decreased need emergency care and re-hospitalization and death. However, when it occurs in isolation, lower results are identified when compared to HV [9].

From this perspective, it remains unclear whether the association of different nursing interventions, such as home visits and telemonitoring, may provide better clinical results than a singular intervention [1,5,6]. Furthermore, there is no systematic review in the literature to evidence this hypothesis. Given the limitations regarding the use of one technique or the other, a systematic review and meta-analysis was conducted to evaluate the effectiveness of a nursing educational intervention using home visits combined with telephone contact in reducing hospital readmission and the mortality of patients with HF, compared to those accompanied by regular health service care, without the combined intervention.

## 2. Materials and Methods

### 2.1. Type of Study

This was a systematic review and meta-analysis of randomized controlled trials (RCT) performed according to the Preferred Reporting Items for Systematic Reviews and Meta-Analyses (PRISMA) recommendations. The systematic review protocol is registered in the International Prospective Register of Systematic Reviews (PROSPERO) CRD42018105760.

### 2.2. Search Strategy and Eligibility Criteria

The review was conducted to answer the research question: “Does the educational intervention provided by a nurse reduce the readmission or the mortality of patients with heart failure?” The PICOS strategy (Population, Intervention, Comparison, Outcome, Study) was used in the following manner: (P) patients diagnosed with HF, (I) educational intervention provided by nurses, (C) comparison with patients without the intervention, (O) primary outcome of readmission, secondary outcome of mortality, and (S) RCT.

The inclusion criteria were: (i) RCT; (ii) patients diagnosed with HF, both clinically and by complementary exams; (iii) at least two groups: control group (CG) and intervention group (IG); (iv) patients from IG receiving a nursing intervention combining HV and telephone contact; (v) outcome variables of readmission or mortality.

The excluded studies included: (i) articles not available in their entirety, even after contacting the authors; (ii) articles in which other health professionals provided the intervention; (iii) an elective outpatient allocation of participants in conditions of clinical stability; (iv) reviews, letters, conference abstracts, editorials, case reports, case series studies, and guidelines. No restriction on language or year of publication was applied.

Detailed search strategies for each of the following bibliographic databases were developed: PubMed/MEDLINE, CINAHL, SciELO and Cochrane. The database search strategies can be found in Appendix A Table A1. A gray literature search included Google Scholar, OpenThesis, and clinical trials. The Google Scholar search was limited to the first 100 most relevant articles published in the last 10 years. The reference lists of all eligible studies and reviews followed the same inclusion steps. Both the gray literature searches and electronic database searches were conducted from their coverage starting date to 7 January 2021. The results obtained were exported to Microsoft Excel™ software version 16.0, with a manual removal of duplicates. Finally, a database was generated with the titles of the articles to be analyzed.

### 2.3. Data Collection

Article selection occurred in two phases. In the first, the titles and abstracts of the articles were analyzed by two researchers (C.R. and Y.A.) in an independent and paired manner. Subsequently, agreement was verified between the examiners regarding the classified references, using the Kappa statistical test. The test defined agreement as low (<0.40), good (0.40–0.75) and excellent (>0.75) [10]. Good or excellent agreement was defined to enable follow-up of the steps. Otherwise, reviewers would have to undergo new training on the subject and eligibility criteria.

When the ideal level of agreement was not obtained, a third reviewer (A.F.M.) was tasked to resolve the issue. The titles and abstracts that met the eligibility criteria were maintained for the second phase. In the second phase, these eligible studies were read in their entirety. The selection system followed the previous step for final decision, and those that were not selected, after discussion, were separately recorded, along with the reason for exclusion. A standardized spreadsheet was developed for data extraction, including the following information from the studies: author(s); year of publication; country; sample of CG and IG; age group; sex; nursing intervention used; follow-up period; number of readmissions; mortality; other main findings; and study limitations. There was no analysis separated by subgroup.

The risk of bias in the included articles was assessed by two independent reviewers (C.R. and Y.A.) using the Cochrane Collaboration’s ROB tool for RCTs and disagreements were resolved by consensus or by a third reviewer (A.F.M.). The risk of bias for each item was individually classified as low, unclear, or high, according to established criteria [11].

### 2.4. Statistical Analysis

A forest plot was used to present the effect sizes and 95% confidence intervals. A two-tailed *p*-value <0.05 was used to determine significance. Statistical heterogeneity was assessed by Cochran’s Q test, and quantified by the I^2^ index. The Cochran Q statistic was used to evaluate the heterogeneity of the studies included in the meta-analysis, and *p* = 0.100 indicated significant heterogeneity. The I^2^ test was used to evaluate the consistency of the effects in studies in which a zero value indicated lack of heterogeneity, I^2^ ≤ 25% indicated low heterogeneity, 25–75% indicated moderate heterogeneity, and ≥75% indicated high heterogeneity. The relative risk (RR) with a 95% CI was calculated for dichotomous outcomes (occurrence of readmission or mortality or lack of occurrence). For results with high or moderate heterogeneity between studies, sensitivity analysis was performed to explore the effect of each individual study on the combined global estimate. In this way, the RR of studies with a low risk of bias to hide the allocation were analyzed and presented in a forest plot. The analysis was performed using the Review Manager, version 5.3 (Cochrane IMS).

## 3. Results

### 3.1. Included Studies

**Figure 1:**
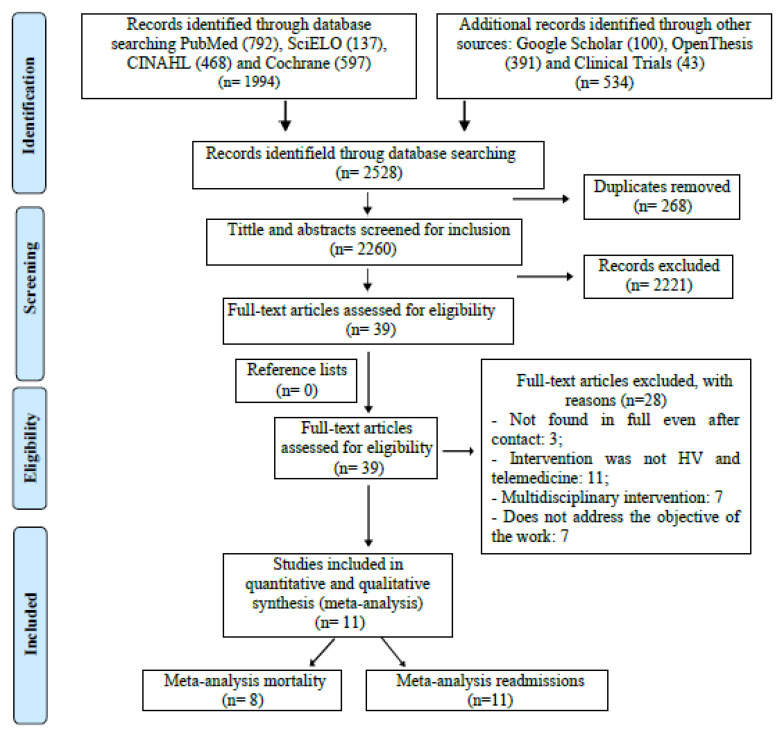
PRISMA flow diagram. PRISMA flow diagram showing study identification, selection, eligibility, and inclusion. Eleven articles were selected for this review. HV: Home Visit.

The process of identification, screening, eligibility, and inclusion is depicted in the PRISMA flowchart (Figure 1). A total of 2528 studies were identified. Of these, 268 studies were duplicates, 2221 studies were excluded based on titles and abstracts, and 28 studies were excluded after screened the full text versions. Thus, 11 articles were selected for this review [12,13,14,15,16,17,18,19,20,21,22]. The kappa test was applied in the first stage of selection, reaching a level of 0.63, which is considered good for continuity of the following stages.

### 3.2. Study Characteristics

The selected articles were published between 2001 and 2019. A total of 1417 patients were analyzed, with 683 in IGs and 734 in CGs. Male participants (56%) and a mean age of 72 years prevailed in IG. CG had a higher participation of men (55%), and a mean age of 74. A summary of the descriptive characteristics of the included articles is provided in Table 1.

The quantity and frequency of home evaluation varied among studies. A total of 73% (n = 8) of the analyzed articles revealed the total number of HV performed, ranging from one to nine, with a mean of four visits. The frequency of visits was not established a priori, as it was influenced by the clinical presentation of the patient post-discharge. The ideal duration of follow-up and the number of visits per year have not been clearly defined and are likely to depend on the patient and clinical characteristics.

Telephone intervention was used as a complementary method to HV in all 11 studies. As for HV, some variation was identified in the number of assessments and the time between each telephone contact. Some authors [16,17,18,19,20,21,22] provided the number of calls made to patients, with a range of 4–18, and a mean of 9 within the follow-up period. The ideal number of telephone consultations and the duration of each call were not defined in this study.

The readmission and mortality results were the primary outcomes analyzed in this review and are shown in Table 1. Different authors evaluated the impact of educational interventions on QoL [16,17,18,21,22], knowledge of the disease [20,22], self-care [17,20,22], costs of readmission for HF [12,19], and the cost-effectiveness ratio of the intervention [12]. The comparison of results between groups in each study is shown in Table 1.

### 3.3. Risk of Bias

In the risk of bias analysis, the kappa coefficient between the two reviewers was considered excellent (0.81). Appendix A Table A2 shows the bias risk analysis for each selected study.

### 3.4. Meta-Analysis for Readmission

**Figure 2:**
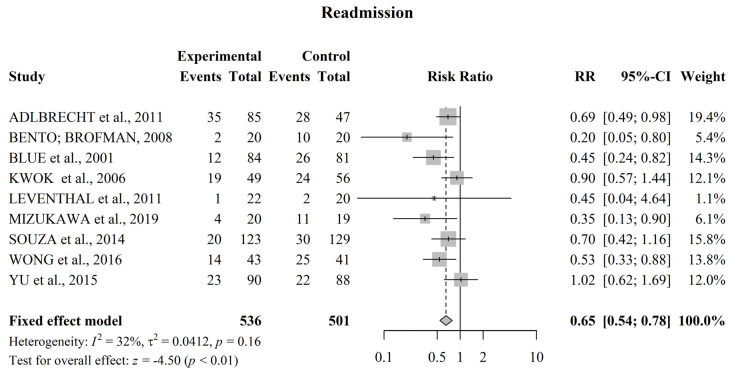
Forest plot of the effect of educational nursing interventions on reducing readmissions in patients with HF. The meta-analysis identified a 36% reduction in the risk of readmission [RR 0.64, 95% CI, 0.54−0.75, *p* < 0.01]. CI = confidence interval; RR = relative risk. References involved in the study: [12,13,14,15,16,17,20,21,22].

Figure 2 shows a meta-analysis of the effectiveness of nursing interventions on reducing readmissions in patients with HF. IG presented a lower number of readmissions in the follow-up period compared to CG (155 vs. 245, respectively), except in two studies [12,16]. Moderate heterogeneity was detected among studies (I^2^ = 39%). The meta-analysis indicated that the proposed educational intervention reduced the risk of readmission by 36% when compared to usual care [RR 0.64, 95% CI, 0.54–0.75, *p* < 0.01].

### 3.5. Meta-Analysis for Mortality

**Figure 3:**
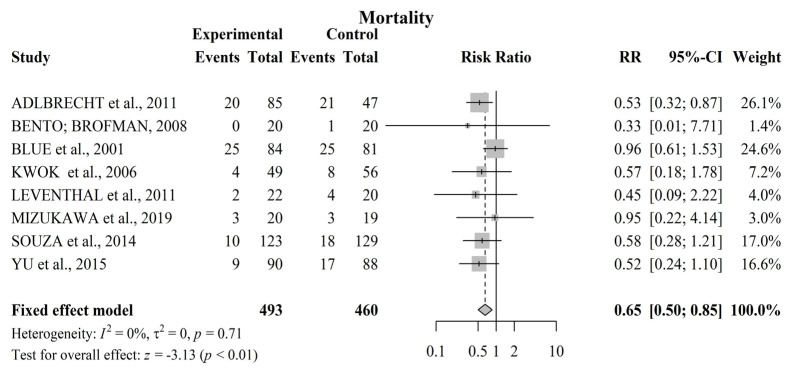
Forest plot of the effect of educational nursing interventions on reducing mortality in patients with HF. The meta-analysis identified a 35% reduction in mortality in the IG [RR 0.65, 95% CI, 0.50−0.85. References involved in the study: [12,13,14,15,16,17,20,22].

Figure 3 shows a meta-analysis of the effectiveness of nursing interventions on reducing the mortality of patients with HF. In this analysis, eight studies presented mortality data [12,13,14,16,17,20,22]. A total of 953 patients participated in the studies that investigated this outcome. Of these participants, IG shows a lower number of deaths in the follow-up period compared to CG (73 vs. 97, respectively), except in tow studies [14,17] in which an equal value was noted between groups. The risk of bias analysis using the Q test expressed by I^2^ indicates that the studies were homogeneous (0%) with regard to their methodological process. The meta-analysis indicates that patients with nursing follow-up supported by a pre-established educational intervention had a 35% lower risk of death when compared to those treated with routine care [RR 0.65, 95% CI, 0.50–0.85, *p* < 0.01].

## 4. Discussion

The main findings of this systematic review and meta-analysis include: (1) educational intervention by nurses, from home visits combined with telephone contact, reduced the chance of readmission by 36% compared to the usual care group; and (2) IG patients showed a 35% reduction in the risk of death when compared to those in CG.

The results of our study are similar to the results of systematic reviews previously published, indicating a lower rate of readmission or mortality in individuals with HF followed by nurses after hospital discharge [23,24]. A meta-analysis carried out by Van Spall and colleagues [23], which included 53 RCT studies, showed 22% less risk of death [RR 0.78, 95% CI, 0.62–0.98] and 35% readmission for all causes [RR 0.65, 95% CI, 0.49–0.86] in IG patients followed by nurses with RV compared to CG with usual care. Slyer and collaborators [24] evaluated studies published until July 2010 in their meta-analysis. They show that the transition from health care intervention by HV and telephone contact led by a nurse can reduce the rate of readmission of patients with heart failure. However, the results were not statistically significant (RR 0.80, 95% CI, 0.66–0.96 and RR 1.06, 95% CI, 0.95–1.19, respectively).

As of today, there is no other known method than meta-analysis that has assessed the impact of nursing intervention with home visits associated with telephone consultations after hospital discharge of patients with HF on readmission or mortality rates. In addition to the primary outcomes mentioned above, our review incorporates evidence from different authors who assessed the impact of educational interventions on QoL, knowledge of the disease, self-care, readmission costs for HF and the cost-effectiveness of the intervention.

Non-adherence to established therapy for symptom control, in addition to sudden clinical changes generated by the disease, require urgent intervention and this is reflected in frequent hospital readmissions [1]. Similarity was found in the aspects addressed in each consultation with the patient in the selected studies, both in the HV and telephone contacts. Although the studies are not exclusively Brazilian, the approach for patients with HF in other countries was similar to the recommendations of the latest Brazilian HF guideline and other international guidelines, such as the European Society of Cardiology, American College of Cardiology/American Heart Association and Canadian Cardiovascular Society, which highlights clinical assessment and support in relation to adequate food, therapeutic adherence and behavioral adjustments to stimulate self-care [8]. This review showed benefits in reducing mortality and readmission when nurses held the following-up of patients after hospital discharge with face-to-face assessments combined with telephone call interventions. However, the ideal duration of post-discharge follow-up and the number of personal assessments and telephone consultations were not defined as our primary outcome. Therefore, a new meta-analysis that includes this objective is suggested.

The meta-analysis carried out by Gandhi et al. [25] showed a reduction in readmission and mortality in the group of patients who were followed up for ≥3 months in a HF clinic after recent decompensation that required a visit to the emergency room or hospitalization. Other studies recommend that the first home visit be carried out at least in the first 14 days after discharge [8] and within the period considered most critical for readmission (first 30 days after discharge) [26].

Before establishing the intervention strategy, health professionals should determine the stage of HF, the functional status verified in previous assessments, the date of the last hospitalization, the patient’s clinical comorbidities, as well as the availability of human and financial resources to perform the follow-up. Ten of the studies selected in this systematic review reported the necessary follow-up time [12,14,15,16,17,18,19,20,21,22], except for Bento and Brofman [13]. This process should be adapted to the reality experienced by patients and their caregivers when moving from a technologically supported environment and with a multidisciplinary team (hospital) to another that does not have essential resources for the continuity of care in the most appropriate way (home).

Hospital readmissions related to HF are considered preventable, and the prevention of this problem remains a major focus of care in the management of patients with HF [27]. However, reduction of hospital readmissions and mortality, as a primary outcome, should not always be considered a positive outcome of an intervention, as the results that facilitate the reduction of hospital hospitalization can be translated into higher mortality rates of patients who decompensated the disease and were unable to access the health service [28,29]. Thus, hospitalization can often provide the opportunity to adjust care [30]. Comorbidities can impact the results and are important considerations before the allocation of patients, in order to avoid bias in the results of the study [11].

Some criteria were used in the studies of this review to reduce the impact on the outcome of the proposed intervention, such as exclusion of patients unable or unwilling to comply with the proposed intervention [13,14,17]; those with severe psychiatric disorders or cognitive impairment [16,17,19,21] and recent acute myocardial infarction or stroke [13,14,16,20]; those in the postoperative period from heart surgery or other scheduled surgery [12,13,14,16,22] or end-stage of HF [18]; those in an acute phase secondary to another comorbidity [20]; those with diseases resulting in reduced life expectancy [13,14,15,16,19]; and those on dialysis as a result of kidney disease [16,19].

The differences in the methodologies of each study did not negatively influence the analysis of this review. The heterogeneity of the articles included for hospital readmission outcome analysis was considered moderate (I^2^ = 39%), and the eight articles were considered homogeneous in terms of mortality analysis (I^2^ = 0%). In addition, a sensitivity analysis was carried out for the primary outcome of readmission, as the studies showed moderate heterogeneity with no substantial change observed in the grouped RR, indicating that no individual study had a considerable influence on the grouped estimate.

In addition to clinical deterioration, other aspects contribute to the probability of hospital readmission and mortality, such as psychosocial and socioeconomic factors [28,29,31]. Consequently, new methods for treating the disease, with consequent improvement in psychosocial aspects, are currently needed [32].

Some RCT show that the lack of follow-up focused on patients’ deficits for disease control, such as knowledge and self-care, results in a greater number of hospital readmissions in the CG [3,33]. A nursing intervention based on recommendations for self-care and the distribution of educational booklets was effective in improving quality of life (QoL) components over six months [34]. These data allow us to understand the importance of educational intervention aimed at controlling multiple factors that lead to hospital readmission, aiming to influence behavioral factors patient related to prevent readmissions, such as therapeutic non-adherence and nutritional imprudence [35].

In another aspect, this review showed that constant hospitalizations have an economic impact both for the patient in maintaining self-care and for the health systems that offer care during hospitalization [12,19]. In this sense, it means that in order to carry to some type of intervention aimed at reducing harmful clinical outcomes for the patient, such as hospital readmission and mortality, the reduction costs during a longer survival time should also be taken into account.

The specificities found around the world in relation to health systems require that caregivers can assist patients with heart failure, considering each scenario to achieve the objectives proposed for this care. In Brazil, which has a unified and free health system (SUS, as per its Portuguese acronym), home care is provided by health professionals who provide services in a defined area for the coverage of these professionals. As for the studies listed in this meta-analysis, the proposed follow-up with HV and telephone contact was carried out by professionals belonging to the hospitals linked to each study, with the assistance of their respective funding agency.

This review has some limitations, such as not establishing the ideal number of HV and telephone consultation, an ideal period of follow-up after hospital discharge and not stratification by groups that may benefit from the proposed intervention. However, the meta-analysis showed effectiveness in reducing the outcomes of readmission and mortality with HV and telephone contact made by nurses.

## 5. Conclusions

Evidence indicates that the use of HV and telephone contact for patient follow-up after hospital discharge for HF decompensation contribute to a reduction in hospital readmission and mortality and is a potential educational strategy for nursing practice. Despite the limitations related to the short follow-up time and small sample size, as well as the absence of pre-established protocols in some of the RCT, the results presented were not compromised. In this review, some studies demonstrated benefits in knowledge, self-care, and QoL outcomes. Further studies of meta-analyses should be performed in order to evidence the cost-effectiveness of the educational intervention proposed in these and other outcomes.

## Figures and Tables

**Table 1:** Main characteristics of the studies eligible for analysis.

Author, Year and Country	Population (IG) (CG)	Sex	Mean Age	Intervention	Readmission/Re-Hospitalization	Follow-Up Time(HV and Telephone Contact)	Mortality	Main Findings	Limitations
Adlbrecht et al., 2011 (Austria) [12]	IG 85 CG 47	♀ 28% & ♂ 71% ♀ 29% & ♂ 70%	72.6 73.7	4 HV: 1°, 3°, 6° & 12° month. Does not specify total or frequency of telephone contact.	IG 35 CG 28	18 months	IG 20 CG 21	- IG with lower outcomes compared to CG: combined outcome of death or re-hospitalization (53% vs. 66%), number of deaths (24% vs. 45%), re-hospitalizations from any cause (75% vs. 83%) and HF (41% vs. 60%), re-hospitalization costs for any reason (12,391 vs. 12,450 euros) and by HF (5225 vs. 7109 euros), cost-effectiveness at one year of survival after discharge from non-HF readmission (0.83 euros/less in the IG) and by HF (26.36 euros/less in the IG).	- Does not report/establish telephone contact number; - Does not report the causes of re-hospitalization related to HF.
Bento; Brofman, 2008 (Brazil) [13]	IG 20 CG 20	♀ 35% & ♂ 65% ♀ 25% & ♂ 75%	54.2 60.6	HV monthly or biweekly as needed. Does not specify the total. Telephone contact biweekly. Does not specify the total.	IG 2CG 10	Not reported	IG 0 CG 1	- IG had a lower number of readmitted patients (2) and a lower frequency of hospitalizations (5) compared to the CG (10 and 22, respectively).	- Small sample. - Does not cite patient follow-up time and does not establish HV number and telephone contact.
Blue et al., 2001 (Scotland) [14]	IG 84 CG 81	♀ 36% & ♂64% ♀ 49% & ♂ 51%	74.4 75.6	HV: Does not specify the total. Telephone contact as needed. Does not specify the total.	IG 12 CG 26	12 months	IG 25 CG 25	- IG with a lower number of readmissions for HF (12 vs. 26) and non-HF (47 vs. 49), length of hospital stay (8 × 9 days) and reduced risk of hospitalization (62%) compared to CG.	- Does not describe HV number and/or telephone contact.
Kwok et al., 2006 (China) [15]	IG 49 CG 56	♀ 55% & ♂ 45% ♀ 55% & ♂ 45%	79.5 76.8	9 HV: weekly in the first month, the first in up to seven days after discharge and monthly in the remaining months. Telephone contacts as needed.	IG 19 CG 24	6 months	CG 4 IG 8	- IG with lower outcomes compared to CG: unplanned readmission (19 vs. 24), health care costs (HK $5229 vs. HK $20,916) and physical limitation (44 m vs. 25 m); - Main cause of readmission was exacerbation of HF symptoms (52%).	- Moderate follow-up time; - Only included patients over 60 years old; - Small sample; - Does not specify the causes of readmissions by analyzed group.
Leventhal et al., 2011 (Switzerland) [16]	IG 22 CG 20	♀ 40% & ♂ 59% ♀ 35% & ♂ 65%	76.7 77.6	1 HV: until 7 days after discharge. 17 Telephone contacts: weekly ×4, bimonthly ×4 and monthly ×6.	IG 01 CG 02	12 months	IG 02 CG 04	- IG had better outcomes in relation to CG: death (2 vs. 4), HF readmission (1 vs. 2) and QoL. - CG had better outcomes in relation to IG in: number of readmissions for any cause (6 vs. 10) and for non-HF cardiac cause (2 vs. 3).	- Small sample; - Number of HV in relation to follow-up time
Mizukawa et al., 2019 (Japan) [17]	IG 20 CG 19	♀ 50% & ♂ 50% ♀47% & ♂ 53%	70.5 74.5	6 HV: mensal. Telephone contacts as needed (162 in total).	IG 4 CG 11	24 months	IG 3 CG 3	- IG had better outcomes in relation to CG: readmissions for cardiac cause (4 vs. 11) and non-cardiac cause (12 vs. 13), patients who had at least one readmission (1 vs. 7), less days of hospitalization (59 vs. 66); - IG improved QoL during follow-up (*p* = 0.002, *p* = 0.012, *p* = 0.003, *p* = 0.018); - IG improved self-efficacy (*p* < 0.001) and self-care behavior (*p* = 0.002).	- Short follow-up time; - Does not describe HV number and/or telephone contact.
Quinn, 2006 (USA) [18]	IG 17 CG 05	♀47% & ♂ 52% ♀ 60% & ♂ 40%	76.5 76.2	8 HV: six in the 1st month, one in the 2nd and 3rd months. 4 telephone contacts: one in the 1st month and three in the 2nd month.	IG 2 CG 4	3 months	Not reported	- IG with report of decreased symptoms of the disease: angina (13.3%), dyspnea (9.7%) and fatigue (33.3%), fewer readmissions (29%) compared to CG (80%) and increase in QoL (from 20% good or excellent to 47%).	- Short follow-up time; - Small sample; - Non-fulfillment of HV strategy and established telephone contact; - Does not report cause of readmission.
Riegel et al., 2002 (USA) [19]	IG 130 CG 228	♀ 46% & ♂53% ♀ 53% & ♂ 46%	72.5 74.6	Total HV and frequency not reported. 17 telephone contacts: the first 5 days after discharge and the others varied.	IG 23 CG 63	6 months	Not reported	- IG with better outcomes in relation to CG in: number of hospitalizations per HF (23 vs. 63), non-HF (56 vs. 114), hospitalization costs (1192 vs. 2186 dollars), HF hospitalized days (1.1 vs. 2.1), non-HF hospitalized days (3.5 vs. 4.8), time to post- discharge hospitalization (129 vs. 116 days), multiple readmissions (17 vs. 52) and satisfaction with care (22.88 vs. 21.66).	- Selective outcome report (satisfaction and use of outpatient resources); - Does not report mortality rate; HV frequency; telephone contact pattern and the causes of readmissions.
Souza et al., 2014 (Brazil) [20]	IG 123 CG 129	♀ 39% & ♂ 61% ♀ 35% & ♂ 64%	62.063.0	4 HV: one in up to 10 days after discharge and the others after 30, 60 and 120 days. 4 telephone contacts. Does not specify frequency.	IG 20 CG 30	6 months	IG 10 CG 18	- IG with better baseline (55%) and final (71%) knowledge about HF in relation to CG (54 and 55, respectively); - IG with better baseline (35%) and final (23%) self-care assessment in relation to CG (34% and 30%, respectively); - IG with lower number of urgent visits (24), readmission for HF (20) or other causes (18) in relation to CG (35, 30 and 19, respectively).	- Does not report the cause of readmission.
Wong et al., 2016 (China) [21]	IG 43 CG 41	♀ 56% & ♂ 43% ♀ 39% & ♂ 61%	78.3 78.4	4 HV: two in the first month, one each week, and monthly in the second and third months. 4 telephone contacts: two in the first month, one each week, monthly in the 2nd and 3rd months.	IG 14 CG 25	3 months	Not reported	- IG with best outcomes in relation to CG: number of readmission in 12 weeks (14 25), intensity of symptoms (23.97 vs. 32.39), functional status in palliative care (66.8 vs. 66.85), QoL (7.57 vs. 6.46), questionnaire on HF (5.26 vs. 4.47) and degree of satisfaction of care (48.84 vs. 36.55).	- Included only patients in palliative care; - Does not report mortality value; - Limited description of the established therapeutic plan.
Yu et al., 2015 (china) [22]	IG 90 CG 88	♀ 46% & ♂ 53% ♀ 63% & ♂ 36%	78.6 78.7	2 HV: one per week in the first month after discharge. 9 phone contacts: one every 2 weeks for 3 months and one every 2 months for 6 months.	IG 23 CG 22	9 months	IG 9 CG 17	- IG had better outcomes than CG in: death (9 vs. 17), better survival without events (67% vs. 63%), total readmission time (229 vs. 408), days of hospitalization (7 vs. 11), self-care (maintenance 53 vs. 40 and self-confidence (39 vs. 26), QoL (70 vs. 59), knowledge (7.9 vs. 6.2), and self-assessment of health (visual scale 67.5 vs. 58, utility index 76 vs. 62) - IG had lower mortality risk (45%).	- Inclusion of patients aged 60 and over; - Does not provide clear data regarding the number of HV; - Does not report causes of readmissions.

HV: home visits; HF: Heart Failure; IG: intervention group; CG: control group; QoL: quality of life; ♀: women; ♂: men. RR: 0.64. The follow-up period after discharge ranged from three to 24 months, with six months (33%) being the most common. During this period, the intervention included HV combined with telephone contact. The follow-up for CG included usual care according to the protocol of each institution at which the patient was admitted (Table 2).

**Table 2:** Strategies intervention versus control.

Intervention Group Care	Control Group Care
Home Visit: - Physical examination with emphasis on the cardiorespiratory system; - Verification of vital signs (blood pressure, heart rate, temperature, respiratory rate and heart rate), peripheral oxygen saturation with pulse oximeter) and anthropometric measurements (weight, height, and body mass index); - Instructions regarding pharmacological treatment (dosage, schedule, indications, contraindications, therapeutic and side effects), clinical manifestations typical of the disease, signs of decompensation, eating habits with emphasis on fluid and salt restrictions and a diet rich in vegetables and fresh fruits, and regular and moderate physical activity. Telephone contact - Complementary method: It was performed with an educational focus, reinforcing instructions provided in person, adjusting errors noted in self-care, monitoring pharmacological compliance, elucidating concerns, registering deaths or readmissions, and scheduling in-person assessments.	Follow-up was carried out with usual care according to the protocol of each institution in which the patient had been admitted. It was not described by the authors. Each countries might have different healthcare systems in this area

## Data Availability

Not applicable.

## Appendix A

**Table A1:** Search strategy used in PubMed, SciELO, CINAHL, Cochrane, Google Scholar, Clinical Trials, and OpenThesis databases.

BASE	Search Strategy
PubMed	(((“Heart Failure”[Mesh] OR “Heart Failure” OR “Cardiac Failure” OR “Heart Decompensation” OR “Heart Failure, Right Sided” OR “Right-Sided Heart Failure” OR “Right Sided Heart Failure” OR “Myocardial Failure” OR “Congestive Heart Failure” OR “Heart Failure, Congestive” OR “Heart Failure, Left Sided” OR “Left Sided Heart Failure”)) AND ((“Patient Education as Topic”[Mesh] OR “Education, Patient” OR “Patient Education” OR “Education of Patients” OR “Self Care”[Mesh] OR “Self-Care” OR “Nursing Care”[Mesh] OR “Nursing Care” OR “Care, Nursing” OR “Management, Nursing Care” OR “Nursing Care Management” OR “educative intervention” OR “educational intervention” OR “nursing intervention”))) AND ((((clinical[Title/Abstract] AND trial[Title/Abstract]) OR clinical trials as topic[MeSH Terms] OR clinical trial[Publication Type] OR random*[Title/Abstract] OR random allocation[MeSH Terms] OR therapeutic use[MeSH Subheading])))
SciELO	((“Heart Failure” OR “Heart Failure, Systolic” OR “Heart Failure, Diastolic”) AND (“Nursing” OR “Nursing Assessment” OR “Nursing Care” OR “Cardiovascular Nursing” OR “Home Health Nursing” OR “Patient Care Planning”) AND (“Patient Education as topic” OR “Patient Education Handout”)) ((“Insuficiência cardíaca” OR “Insuficiência Cardíaca sistólica” OR “Insuficiência Cardíaca diastólica”)) AND ((enfermagem OR “Avaliação em Enfermagem” OR “Cuidados de Enfermagem” OR “Enfermagem Cardiovascular” OR “Enfermagem Domiciliar” OR “Planejamento de Assistência ao Paciente”)) AND ((“Educação de Pacientes como Assunto” OR “Prospecto para Educação de Pacientes”)) ((“Insuficiencia Cardíaca” OR “Insuficiencia Cardíaca Sistólica” OR “Insuficiencia Cardíaca Diastólica” AND (Enfermería OR “Evaluación en Enfermería” OR “Atención de Enfermería” OR “Enfermería Cardiovascular” OR “Cuidados de Enfermería en el Hogar” OR “Planificación de Atención al Paciente”) AND (“Educación del Paciente como Asunto” OR “Folleto Informativo para Pacientes”))
CINAHL	((MM “Heart Failure”) OR (MM “Heart Injuries”) OR (MM “Heart Hypertrophy”) OR (MM “Heart”) OR (MM “Heart Diseases”) OR (MH “Coronary Disease”) OR (MM “Cardiac Patients”) AND (MM “Education, Nursing, Associate”) OR (MM “Practical Nursing”) OR (MM “Nursing Protocols”) OR (MM “Nursing Interventions”) OR (MM “Nursing Care Plans”) OR (MM “Education, Nursing, Practical”) OR (MH “Nursing Practice”) AND (MM “Patient Education”) OR (MM “Patient Discharge Education”) OR (MM “Cardiac Patients”) OR (MM “Patient Orientation”))
COCHRANE	(“Heart Failure” OR “Heart Failure” OR “Cardiac Failure” OR “Heart Decompensation” OR “Heart Failure, Right Sided” OR “Right-Sided Heart Failure” OR “Right Sided Heart Failure” OR “Myocardial Failure” OR “Congestive Heart Failure” OR “Heart Failure, Congestive” OR “Heart Failure, Left Sided” OR “Left Sided Heart Failure”) AND (“Patient Education as Topic” OR “Education, Patient” OR “Patient Education” OR “Education of Patients” OR “Self Care” OR “Self-Care”) AND (“Nursing Care” OR “Nursing Care” OR “Care, Nursing” OR “Management, Nursing Care” OR “Nursing Care Management” OR “educative intervention” OR “educational intervention” OR “nursing intervention”)
Google Scholar	(“Heart Failure”) and (“Nursing” or “Patient Education”)
Open Thesis	((“Heart Failure”) AND (“Nurse” OR “Nursing” OR “Nurses”) AND (“Patient education” OR “Self-care”))
Clinicaltrials.gov	“Nursing” and “Heart Failure” and “Patient Education”

**Table A2:** Risk of bias assessment.

Authors	Random Sequence Generation	Allocation Concealment	Blinding of Participants and Professionals	Blinding of Outcome Evaluators	Incomplete Outcomes	Selective Outcome Report	Other Sources of Bias
Adlbrecht et al., 2011 [12]							
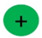
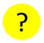
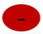
Bento; Brofman, 2008 [13]							
Blue et al., 2001 [14]							
Kwok et al., 2006 [15]							
Leventhal et al., 2011 [16]							
Mizukawa et al., 2019 [17]							
Quinn, 2006 [18]							
Riegel et al., 2002 [19]							
Souza et al., 2014 [20]							
Wong et al., 2016 [21]							
Yu et al., 2015 [22]							

(+) low risk of bias, (-) high risk of bias, (?) Risk of uncertain bias.
